# The Therapeutic Potential of Cefiderocol in the Treatment of Multidrug-Resistant Gram-Negative Bacteria: A Narrative Review

**DOI:** 10.3390/jcm14238415

**Published:** 2025-11-27

**Authors:** Aleksandra Złotowska, Wiktoria Hanna Buzun, Karolina Pełka, Zuzanna Zalewska, Wiesława Duszyńska

**Affiliations:** 1 The Students Scientific Association by Department and Clinic of Anaesthesiology and Intensive Therapy, Faculty of Medicine, Wroclaw Medical University, L. Pasteura Street 1, 50-367 Wroclaw, Poland; aleksandra.zlotowska@student.umw.edu.pl (A.Z.); wiktoria.buzun@student.umw.edu.pl (W.H.B.); karolina.pelka@student.umw.edu.pl (K.P.); zuzanna.zalewska@student.umw.edu.pl (Z.Z.); 2 Department and Clinic of Anaesthesiology and Intensive Therapy, Faculty of Medicine, Wroclaw Medical University, L. Pasteura Street 1, 50-367 Wroclaw, Poland

**Keywords:** cefiderocol, MDR, Gram-negative bacteria, β-lactam antibiotics, siderophore cephalosporin, antimicrobial resistance

## Abstract

**Background:** Increasing antimicrobial resistance (AMR) is one of the leading causes of death worldwide. The predominant pathogens that exacerbate the AMR problem are multidrug-resistant (MDR) Gram-negative bacteria (GNB). Due to the increasing adaptation of MDR GNB to commercially available antimicrobial drugs, such as carbapenems as well as third- and fourth-generation cephalosporins, pharmaceutical companies around the world have been forced to produce increasingly innovative chemotherapeutics. Cefiderocol (CFDC) is a novel injectable cephalosporin 5 generation developed by Shionogi, directed against MDR GNB, including strains resistant to carbapenems. **Results:** Analysis demonstrated its significant efficacy across a wide range of in vitro and in vivo studies against MDR GNB, including Carbapenem-resistant *Pseudomonas aeruginosa* (CRPA), Carbapenem-resistant *Acinetobacter baumannii* (CRAB), and carbapenem-resistant Enterobacterales (CRE) (WHO Critical Priority Pathogens). Clinical studies have shown CFDC to be an effective drug with few adverse effects. **Conclusions:** When used CFDC appropriately within antibiotic stewardship guidelines, this drug is an effective, well-tolerated targeted treatment option for patients with severe clinical conditions.

## 1. Introduction

Antimicrobial resistance (AMR) has been considered by the WHO as one of 10 public health threats since 2019 [1]. Increasing AMR is one of the leading causes of death worldwide. The predominant pathogens that exacerbate the AMR problem include both multidrug-resistant (MDR) Gram-negative bacteria (GNB) and MDR Gram-positive organisms, such as methicillin-resistant *Staphylococcus aureus* (MRSA) and vancomycin-resistant enterococci (VRE) [2].

The European Centre for Disease Prevention and Control (ECDC) Rapid Risk Assessment Update III on CRE, published in 2025, highlights the risks associated with the further spread of CRE in the European Union/European Economic Area (EU/EEA) [3]. Data from the European Antimicrobial Resistance Surveillance Network (EARS-Net) in 2023 revealed a notable increase in the percentage of *Klebsiella pneumoniae* (*K. pneumoniae*) isolates resistant to carbapenems from invasive bloodstream infections within the EU between 2019 and 2023, reaching 57.5% [3,4]. An upward trend was also observed for carbapenem-resistant *Pseudomonas aeruginosa* (*P. aeruginosa*) [4]. In addition, in 36% of European countries, the percentage of resistance to carbapenems was higher for *Acinetobacter* spp. and *P. aeruginosa* than for *K. pneumoniae* [5]. In the case of resistance to third-generation cephalosporins, only 7 of 44 European Countries reported *K. pneumoniae* resistance rates of less than 10%, while as many as 19 countries had resistance rates of more than 50% [5]. In contrast, the prevalence of bloodstream infections caused by *Escherichia coli* (*E. coli*) resistant to third-generation cephalosporins did not show a significantly static difference between 2023 and 2019, and was 10.35 per 100,000 population in 2023 in the EU [4].

In the situation of resistance to third- and fourth-generation cephalosporins caused by extended-spectrum β-lactamases (ESBL), carbapenems and aminoglycosides remained the pharmaceuticals most commonly used in MDR infections [6,7,8]. However, the emergence of resistance to carbapenems, due in part to the increased prevalence of metallo β-lactamase (MBL)-producing bacteria, has resulted in an escalation of antibiotic resistance, posing a serious public health threat [9,10,11]. MBLs have zinc in their active center, making them capable of hydrolyzing all β-lactam antibiotics, except aztreonam [12,13]. Consistently, MDR GNB strains such as carbapenem-resistant *A. baumannii* (CRAB), carbapenem-resistant *K. pneumoniae* (CRKP), carbapenem-resistant Enterobacterales Oxa-48 (CRE-OXa-48) and carbapenem-resistant Enterobacterales EMBL (CRE -EMBL) have emerged worldwide [14].

It is worth emphasizing that infections caused by strains of GNB resistant to third-generation cephalosporins and resistant to carbapenems such as *Acinetobacter* spp., *K. pneumoniae* or *P. aeruginosa* DTR (difficult-to-treat resistance) are associated with increased mortality [4,15,16,17]. According to the ECDC, in 2015, MDR GNB infections were responsible for 70% of infection-related deaths [18]. According to the EPIC (Extended Surveillance on Prevalence of Infection in Intensive Care) III study, 30% of patients with confirmed infection died during hospitalization [19]. Further analysis showed that Klebsiella infections resistant to β-lactam antibiotics, including cephalosporins and carbapenems, as well as carbapenem-resistant Acinetobacter were associated with a higher risk of death than infections caused by other microorganisms [20].

Due to the increasing adaptation of MDR GNB to commercially available antimicrobial drugs, such as carbapenems as well as third- and fourth-generation cephalosporins, pharmaceutical companies around the world have been forced to produce increasingly innovative chemotherapeutics. Since the WHO report, published in 2017 [21] and concerning the need to find new drugs against priority pathogens (e.g., CRAB, CRE, CRPA, CRKP), by 2020 7 new antibiotics have been registered by the European Medicines Agency (EMA) [22]. One of them is cefiderocol (CFDC).

CFDC, a bactericidal cephalosporin with siderophore-like properties that uses active iron transport to penetrate GNB, has significant therapeutic potential against infections caused by β-lactamase producing bacteria including MBL. CFDC is used to manage complicated urinary tract infections (UTIs); hospital-acquired pneumonia (HAP) in non-ventilated patients and ventilator-associated pneumonia (VAP), healthcare-associated pneumonia; in patients with bacteraemia and septicaemia; and in other infections as rescue therapy [15,23,24]. Clinical data on the efficacy of CFDC are still inconclusive and require further analysis, as well as data about increasing resistance which need consistent attention [24,25].

MDR-associated infections pose a significant burden on the healthcare system, both because of the serious health risks to patients and for economic reasons [25,26]. With this in mind, we decided to conduct a narrative review examining the potential of CFDC, highlighting its promising therapeutic benefits resulting from its high potency against MDR GNB pathogens. Its effectiveness and relative novelty on the market have enabled progress in addressing the growing challenge of antimicrobial resistance.

## 2. Antibiotic Resistance Mechanisms in Gram-Negative Bacteria in Relation to Potential Activity of Cefiderocol

Bacteria have developed various mechanisms of antibiotic resistance, which can be classified into four main strategies, including drug inactivation, bacterial target site modifications, limiting drug uptake, and high levels of drug efflux [27].

A mechanism of great importance in the development of antibiotic resistance is the production of bacterial enzymes, which increase bacterial survival in the presence of anibiotics [28]. Regarding the potential activity against CFDC, the most important are β-lactamases whose action involves hydrolytic cleavage of the β-lactam ring, leading to the inactivation of the antibiotic [29]. These enzymes have been divided by Ambler into 4 classes (A, B, C, D) based on the differences in their amino acid sequence [29,30]. However, thanks to its structure, CFDC exhibits intrinsic stability and is resistant to hydrolysis by these enzymes, including KPC, AmpC, ESBL+, MBL carbapenemases, as well as NDM, IMP, and VIM [30,31,32]

Moreover, bacteria have developed mechanisms that enable them to limit the influx and increase the efflux of antibiotics from the cell [33]. Hydrophilic substances, such as β-lactams, need special transport proteins, porins, to enter the cell [34]. Therefore, changes in the permeability of these proteins can significantly reduce the effectiveness of antibiotics [33,34]. In addition, other proteins in the bacterial cell membrane, such as efflux pumps, actively pump antibiotics out of the cell [26]. This reduces the concentration of the antibiotic inside the cell, allowing the bacteria to survive despite the presence of the drug in the external environment [35]. However, in the context of CFDC, the above-mentioned resistance mechanisms are ineffective due to a mechanism of penetration into the bacterial cell that is independent of porins and efflux pumps [31]. This mechanism, often referred to as the “Trojan horse” strategy, is enabled by an additional chlorocatechol group in the antibiotic structure, which gives it siderophore (iron-chelating) activity [32]. Thanks to this characteristic, the antibiotic is transported across the outer membrane of GNBs by natural iron transportation systems [36,37,38].

## 3. Chemical Structure and Mechanism of Action of Cefiderecol

CFDC is a novel injectable cephalosporin 5 generation developed by Shionogi, directed against MDR GNB, including strains resistant to carbapenems [39]. The chemical structure of CFDC is analogous to ceftazidime and cefepime, which belong to the group of 3rd and 4th generation cephalosporins [40,41]. In its structure, a pyrrolidine group is present in the side chain at position 3, as in cefepime, and a carboxypropanoxyimine group in the side chain at position 7 of the cefem nucleus, as in ceftazidime [41,42]. Their presence in the CFDC molecule facilitates pore permeation, conditions resistance to a large number of β-lactamases and intensifies intrinsic activity compared to other extended-spectrum cephalosporins [41]. CFDC is distinguished from ceftazidime and cefepime by the presence of a catechol group in the side chain at position 3, which further enhances its stability against β-lactamases, including carbapenemases, and provides siderophore activity [43,44]. This modification simultaneously preserves the high affinity of the molecular target, penicillin-binding proteins (PBPs) [45].

The transport of CFDC into bacterial cells is accomplished through porin channels and through the active iron transport system by forming complexes with its trivalent ions. This leads to high concentrations of CFDC in the periplasmic space [46]. Penetration through the bacterial cell wall by facilitated (siderophore) and passive/facilitated diffusion (through pores) contributes to partial inhibition of β-lactamases [47]. In the periplasmic space, after release from the complex with iron, the cephalosporin fragment binds to PBP-3, resulting in the inhibition of bacterial cell wall synthesis [48].

## 4. Registered Clinical Indications of Cefiderocol

In 2019, CFDC was approved by the U.S. Food and Drug Administration (FDA) for the treatment of complicated UTIs, and a year later also for the treatment of VAP and HAP [49].

In 2020, the EMA registered CFDC for the treatment of difficult-to-treat infections caused by Gram-negative aerobic bacteria at adult patients when possibility to treatment are limited [50].

## 5. Cefiderocol Dosage According to the Food and Drug Administration (FDA)

### 5.1. Patients with Creatinine Clearance (CLcr) 60–119 mL/min

For patients with a CLcr of 60 to 119 mL/min, the recommended dosage of CFDC sulfate toxylate is 2 g administered every 8 h over a 3 h intravenous infusion. The duration of therapy is 7–14 days, and should be adjusted according to the patient’s clinical condition [51].

### 5.2. Patients with Clcr < 60 mL/min or Patients Receiving Intermittent Hemodialysis (HD)

For patients with CLcr < 60 mL/min and patients undergoing intermittent hemodialysis (HD), the recommended dosage is shown in Table 1. Dosing in patients undergoing intermittent HD should be started immediately after the procedure as it is broken down by HD. For patients with variable renal function, the dose should be monitored and adjusted on an individual basis. Regardless of creatinine clearance, the drug is approved for administration as a 3 h intravenous infusion [52].

### 5.3. Patients Receiving Continuous Renal Replacement Therapy (CRRT)

In patients undergoing continuous renal replacement therapy (CRRT), the dosage of CFDC depends on the effluent flow rate in CRRT. In some cases, it is necessary to adjust the dosage for residual renal function and the patient’s clinical condition. Particularly in the first 24 h of treatment, the patient should be monitored, as it may be necessary to modify the dose of the drug [53]. Recommended dosages are shown in Table 2.

### 5.4. Patients with Clcr ≥ 120 mL/min

In patients with Clcr ≥ 120 mL/min, the recommended dosage is 2 g every 6 h in a 3 h intravenous infusion [46,54].

### 5.5. Special Cases

There are no safety data on the use of CFDC in pregnant women. There are animal studies that do not indicate negative effects of CFDC on pregnancy [55]. Although the available data from prospective cohort studies do not indicate an association between the use of cephalosporins in pregnancy and the miscarriage or occurrence of major birth defects in children, there are no sufficient data on its safety, so it is currently recommended not to use it during pregnancy [56].

There are also no data on whether CFDC is secreted into breast milk. In animal studies, it has been shown to be present in breast milk, which may suggest a similar relationship in humans, but these results are not confirmed. For breastfeeding women, the profit and loss calculus of CFDC use and breastfeeding for the child should be evaluated [57].

For children under 18 years of age, the safety and efficacy of CFDC have not been determined. For the elderly population and people with hepatic impairment, it is not necessary to adjust the dose of the drug.

### 5.6. ECMO

In critically ill patients on extracorporeal membrane oxygenation (ECMO), sequestration/adsorption of pharmaceuticals can occur, which can result in increased or decreased patient exposure to the drug and, in the case of antibiotics, treatment failure and escalation of infection [58]. For this reason, ECMO patients are a particular group in which the patient should be monitored and doses of drug products should be adjusted according to the clinical condition.

In the case of CFDC, ex vivo studies suggest that it does not undergo sequestration or adsorption in patients under ECMO [59,60], but exact data indicating optimization of dosing are yet not available. There are studies suggesting the benefit of using CLcr-dependent doses of CFDC also in patients under ECMO, but in long-term infusion [61]. This is justified, due to the fact that the clinical efficacy of β-lactams depends on the time at which the drug concentration in the blood is higher than minimum inhibitory concentration (MIC) [62]. However, these results need to be confirmed in randomized trials with a higher statistical power.

## 6. Adverse Events of Cefiderocol

CFCF has a high safety profile and few side effects. The most common side effects include gastrointestinal disturbances (diarrhea, vomiting, nausea, constipation), cough, rash, oral yeast infections, increased liver enzymes, and increased creatinine [63].

## 7. Pharmacokinetics of Cefiderocol

CFDC is excreted mainly via the kidneys (98.6%) and has a half-life of 2 to 3 h [64]. In a study by T. Katsube et al. [65] conducted on subjects with renal impairment or end-stage renal failure, reduced clearance and prolonged half-life of CFDC were observed. Maximum plasma concentrations (Cmax) were comparable in the renal impairment and normal renal function groups. The distribution volume values reached approximately 13 L [65].

Its use in the treatment of pneumonia is possible due to its efficient intrapulmonary penetration. It was confirmed in a group of healthy subjects by T. Katsube et al. [66] in a study evaluating the intrapulmonary pharmacokinetics of CFDC following a single dose of 2000 mg as an hourly intravenous infusion. CFDC concentrations in epithelial lining fluid (ELF) at 1, 2, 4 and 6 h after a single intravenous dose of CFDC (2000 mg infused over 60 min) were recorded as 13.8, 6.69, 2.78 and 1.38 mg/L, respectively [66]. Analogous ELF concentrations were obtained for other β-lactams used in respiratory tract infections in a study by K. A. Rodvold [67]. The values of the geometric mean concentration ratios over 6 h ranged from 0.0927 to 0.116 for ELF to total plasma and from 0.00496 to 0.104 for alveolar macrophages (AMs) to total plasma. The AUC ratios of ELF and AMs to total and free plasma were 0.101 and 0.239, respectively. The penetration rate of CFDC to ELF was comparable to ceftazidime in critically ill patients (0.229 based on free plasma using an unbound protein fraction of 0.9). The study showed that CFDC penetrates into ELF and ELF and plasma concentrations appear to parallel each other [66].

## 8. Spectrum of Activity of Cefiderocol

CFDC has a unique and broad spectrum of activity, mainly against aerobic GNB. In vitro studies have shown that a minimum inhibitory concentration (MIC) of CFDC ≤ 2 μg/mL has been reported not only for bacteria belonging to the Enterobacteriaceae family, including *Enterobacter* spp., *E. coli*, *Klebsiella* spp., *Proteus* spp., *Providencia* spp., but also for non-fermenting GNB such as *Acinetobacter* spp., *Pseudomonas* spp. and *Burkholderia* spp. [68,69]. Additionally, CFDC has been shown to be effective in vitro against respiratory pathogens such as *Haemophilus* spp., *Moraxella catarrhalis* and *Bordetella parapertussis* [70].

Against clinically relevant Gram-positive bacteria, such as *Staphylococcus aureus* and *Enterococcus faecalis*, CFDC has limited bactericidal activity [71]. According to a study by A. Ito et al. the MIC value for these pathogens can be as low as ≥32 μg/mL, suggesting a high level of resistance to this antibiotic [70].

However, the bactericidal efficacy of CFDC against some bacterial species remains limited. Examples are *Campylobacter jejuni* and *Neisseria gonorrhoeae*, of which the MICs exceed 4 μg/mL, which is associated with their low sensitivity to CFDC treatment [70].

Despite the high efficacy of CFDC against selected MDR GNB, its activity against anaerobic and Gram-positive bacteria is highly variable. According to the results presented by A. Ito et al. the MIC value for these microorganisms ranges widely from ≤0.031 μg/mL to >32 μg/mL, with the lowest values (≤0.031 μg/mL) recorded for *Fusobacterium necrophorum* [70,71]. Importantly, the MIC values for CFDC against these bacteria are significantly higher than for other β-lactams such as cefepime or meropenem, which significantly limits its use in treating infections caused by these pathogens [71].

According to product characteristics of CFDC, this antibiotic is not recommended for the treatment of anaerobic as well as GPB infections. So in empirical treatment, when GPB infections or anaerobic infection are suspected, antibiotics active for such pathogens should be added to CFDC [72].

### 8.1. Carbapenem-Resistant Pseudomonas aeruginosa

Unlike most new β-lactam antibiotics, CFDC preserves its activity against CRE [73]. Results from the CREDIBLE-CR study suggest that the efficacy of CFDC is comparable to therapies used to treat CRPA infections, such as prolonged infusion of meropenem, polymyxins and aminoglycoside [74]. Therefore, CFDC may be an alternative to polymyxins in the treatment of infections caused by multiresistant *P. aeruginosa* [73].

### 8.2. Carbapenem-Resistant Enterobacterales

CFDC shows high activity against GNB with resistance to carbapenems, including strains producing ESBLs and carbapenemases. In particular, it should be noted that it remains effective against NDM-1 producing *K. pneumoniae*, for which the MIC is ≤8 μg/mL, making it more effective than other antibiotics [73,74]. In comparison, the MICs of amikacin and ciprofloxacin for these pathogens are ≥16 μg/mL. In contrast, only colistin shows a lower MIC (≤1 μg/mL) [73].

### 8.3. Carbapenem-Resistant Acinetobacter baumannii

In numerous in vitro studies, the MIC value of CFDC against *A. baumannii* was <4 mg/mL [73,75,76]. However, results from clinical trials, including CREDIBLE-CR, have shown unsatisfactory therapeutic effects of this antibiotic [74]. The data presented in this paper suggest that the use of CFDC may be associated with poorer clinical outcomes compared to polymyxin-based treatment regimens in the treatment of infections caused by carbapenem-resistant strains of *A. baumannii* [73,74]. In addition, a study published by R.K. Shields et al. [77] revealed a significant prevalence of hetero resistance to CFDC among *A. baumannii* strains, which may explain the limited clinical activity of this antibiotic in the treatment of infections caused by this pathogen [77]. As a result, caution is recommended when using CFDC in monotherapy for CRAB infections and considering combination therapy or alternative treatment regimens [73,77].

## 9. Clinical Trials Conducted Before the Registration of Cefiderocol

Pre-registration studies have shown that CFDC has promising activity against multidrug-resistant Gram-negative bacteria [78,79,80].

R. G. Wunderink et al. [78] conducted a randomized, double-blind, parallel phase 3 study (APEKS-NP) comparing the efficacy of CFDC (*n* = 148) versus high-dose meropenem (*n* = 152) in prolonged infusion in adults with hospital-acquired pneumonia. The percentage of overall mortality at day 14 was 12.4% for CFDC (18/145) and 11.6% for meropenem (17/146); adjusted treatment difference 0.8%, 95%CI: 6.6–8.2, *p* < 0.05) for the no inferiority hypothesis). The frequency of reported adverse events was similar in both groups (88% CFDC, 86% meropenem). The results of the study highlighted that CDFC has similar efficacy to meropenem in treating patients with pneumonia caused by nosocomial bacteria, including those caused by MDR GNB [78].

M. Bassetti et al. [79], in a randomized, open-label, multicenter patient study CREDIBLE-CR, evaluated the efficacy and safety of CFDC (*n* = 80) compared with best available therapy (*n* = 38) in adults with serious carbapenem-resistant Gram-negative infections (nosocomial pneumonia, BSI or septicemia or complicated UTIs). Among the microbial carbapenem-resistant intention-to-treat (ITT) population (118 patients), clinical cure of hospital-acquired pneumonia was achieved by 20/40 patients (50%), 95% CI: 33.8–66.2 in the CFDC group and 10/19 patients (53%), 95% CI: 28.9–75.6 in the best available therapy group. Microbiological eradication was reported in 9/17 (53%) patients with complicated UTIs, 95% CI: 27.8–77.0 in the CFDC group and 1/5 (20%) patients, 95% CI: 0.5–71.6 in the best available therapy group. Adverse events were observed more frequently in the best available therapy group (96%) than in the CFDC group (91%). The study demonstrates that CFDC is an effective choice for the treatment of carbapenem-resistant infections in patients with limited treatment options [79].

S. Portsmouth et al. [80], in a multicenter, double-blind, phase 2 study (APEKS cUTI), evaluated the efficacy and safety of CFDC (*n* = 252) compared with imipenem–cilastatin (*n* = 119) for the treatment of complicated UTIs in patients exposed to MDR GNB. Clinical cure was achieved in 183/252 (73%) of patients in the CFDC group and 65/119 (55%) of patients in the imipenem–cilastatin group, with an adjusted treatment difference of 18.58% (95%CI: 8.23–28.92; *p* < 0.05). Adverse events were reported more frequently in the imipenem–cilastatin group (51%) than in the CFDC group (41%). The study concluded that CFDC is a potential treatment for complicated UTIs in patients with MDR Gram-negative infections, due to its efficacy and safety profile [80].

## 10. The Place of Cefiderocol in Current Guidelines for the Treatment of Infections with Antibiotic-Resistant GNB 

In the last year, several guidelines under the auspices of professional societies like Clinical Microbiology and Infectious Diseases (ESCMID), the Infectious Disease Society of America (IDSA), the Spanish Society of Infectious Diseases and Clinical Microbiology (SEIMC), Italian Society of Infectious and Tropical Diseases (SIMIT), and the French Society of Infectious Diseases (SPILF) which enumerated CFDC as a treatment options for MDR Gram-Negative infections were published [81,82,83,84]. Both the ESCMID 2021 and SEIMC 2022 guidelines were estab lished performing a systematic review of the literature. IDSA 2022 guideline was prepared on a basis of literature non-systematic review and panellist clinical experience, whereas the SEIMC guidelines on a basis literature critical review, IDSA recommendation and greater contribution panellist/expert opinion [81,83,85]. In the SIMIT and SPILF guideline 2023 a panel of experts aimed to address unresolved ESCMID and IDSA issues in clinical practice based on their experience, updated literature review, and open discussions [84].

According to ESCMID guidelines by M. Paul at al. [81] published in 2021, treatment with CFDC (strength of recommendation—conditionally, level of evidence—low) is conditionally recommended for patients with severe infections due to CRE carrying/metallo–β-lactamases and/or resistant to all other antibiotics, including ceftazidime–avibactam and meropenem–vaborbactam [81]. The same guidelines do not recommend combination therapy for patients with CRE infections susceptible to and treated with CFDC, ceftazidime–avibactam or meropenem–vaborbactam (strength of recommendation—strong, level of evidence—low) [81,86].

In patients with severe infections due to difficult-to-treat CRPA, ESCMID guidelines underlined that insufficient evidence is available for CFDC and also imipenem–relebactam, ceftazidime–avibactam at this time. For the treatment CRPA, ceftolozane–tazobactam (if active in vitro) is suggested (strength of recommendation—conditional, level of evidence—very low). Regarding recommendations for the choice of antibiotic treatment for CRAB on the basis of CREDIBLE trial results, the authors of this guideline conditionally recommend against CFDC for the treatment of these infections (strength of recommendation (conditionally—low, level of evidence—low). For the treatment of CRAB infections ampicillin–sulbactam is suggested (if active for sulbactam in vitro). In the summary shown in the table of this guideline, CFDC has potential in vitro activity against target carbapenem-resistant Gram-negative bacteria for CRAB, ESBLs, CRPA non-MBL, CRE non-CP, CRE KPC, CRE OXA 48, and CRE MBL [79,81,87].

Referring to the American Infectious Diseases Association IDSA guidelines by P.D. Tamm et al. [82] published in 2024, CFD is recommended for the treatment pathogens such as ESBLs, AmpC, CRE, *P. aeruginosa* with difficult-to-treat resistance (DTR *P. aeruginosa*), CRAB, and *S. maltophilia* [82].

Although the newer β-lactam–β-lactamase inhibitor combinations and CFDC are expected to be effective against extended-spectrum β-lactamase-producing Enterobacterales (ESBL-E) infections, the panel suggests that these agents should be preferentially reserved for treating carbapenem-resistant organisms or polymicrobial infections including organisms exhibiting carbapenem resistance (e.g., ceftolozane–tazobactam for coinfection with DTR *P. aeruginosa* and ESBL-E) [82,88,89].

Regarding CFDC as a treatment option for AmpC-producing Enterobacterales (AmpC-E infections), CFD is likely to be effective in clinical practice against AmpC, although some case reports indicate the potential for AmpC-E to develop resistance to this antibiotic [82,90]. CFDC, ceftazidime–avibactam, meropenem–vaborbactam, imipenem–cilastatin-relebactam, and are alternative options for uncomplicated CRE cystitis and preferred antibiotics for the treatment of pyelonephritis or cUTI caused by CRE. CFDC is also indicated as an alternative option for the treatment of infections outside of the urinary tract caused by CRE with KPC production [82,91,92].

CFDC is suggested as an alternative agent for treating KPC-producing pathogens due to limited clinical outcomes data and to reserve it for the treatment of infections caused by MBL-producing Gram-negative bacteria [82,93]. Ceftazidime-avibactam in combination with aztreonam, or CFDC as monotherapy, are preferred treatment options for NDM and other MBL-producing Enterobacterales infections outside of the urinary tract caused by CRE. A second preferred option for the treatment of NDM and other MBL-producing Enterobacterales is CFDC [82,94,95]

Ceftolozane–tazobactam, ceftazidime–avibactam, imipenem–cilastatin–relebactam, and CFDC are the preferred treatment options for uncomplicated cystitis caused by DTR *P. aeruginosa*. Simillary, CFDC is an alternative treatment option for infections outside of the urinary tract caused by DTR *P. aeruginosa* [82,87,92,96].

It is worthy to underline that combination antibiotic therapy is not suggested for infections caused by DTR *P. aeruginosa* if susceptibility to ceftolozane–tazobactam, ceftazidime–avibactam, imipenem–cilastatin–relebactam, or CFDC has been confirmed [97].

CFDC should be limited to the treatment of CRAB infections refractory to other antibiotics or in cases where/if intolerance or resistance to other agents is present. When CFDC is used to treat CRAB infections, the panel suggests using it with caution and prescribing it as part of a combination regimen. The panel also suggests limiting consideration of CFDC for CRAB infections after other regimens have been exhausted [82,98].

CFDC is also recommended for the treatment of infections caused by *S. maltophilia* infections as a component of combination therapy [82,99].

Analyzing the SEIMIC guideline published by Pintado V at al. it is possible to find recommendation for CFDC in the treatment of infections caused by Enterobacterales CR, *Pseudomonas* MDR, DTR, *A. baumannii* and *S. maltophilia* [100]. As regards recommendation for Enterobacterales with the different types of carbapenemase SEIMC guidelines agree (with earlier guidelines) on the use of cetazidime–avibactam or meropenem–vaborbactam just for KPC producers, and on the use of ceftazidime/avibactam for OXA-48 producers and in combination with aztreonam for MBL producers. Similarly, CFDC is recommended for MBL infection treatment [100,101].

For non-serious and low-risk *P. aeruginosa* MDR, DTR infections SEIMC guideline preferred recommendation is ceftolozane–tazobactam and as an alternative ceftazidime–avibactam or CFDC. For severe cases, SEIMC’s preferred recommendation is ceftolozane–tazobactam and as an alternative ceftazidime–avibactam, imipenem–relebactam, colistin or CFDC [82,83,100].

In mild-to-moderate infections caused by ampicillin-resistant *A. baumannii*, there are relevant differences between different guidelines but only SEIMC recommend CFDC in combination with colistin or a triple therapy for *A. baumannii PDR* (pandrug-resistance) infections [83,100,102].

In severe *A. baumannii* infections, the SEIMC guideline recommends to use CFDC as part of combination therapy but to avoid rifampicin in combination [100,103].

For mild *S. maltophilia* infection, the first-line recommendation is co-trimoxazole and minocycline in monotherapy, and second tigecycline, levofloxacin or CFDC in monotherapy. For moderate-to-severe *S. maltophilia* infection, CFDC is recommended as a combined therapy with co-trimoxazole if after initiation of co-trimoxazole in monotherapy, there is delay in clinical improvement. Preferred agents for combined therapy in this case is minocycline, but tigecycline, levofloxacin or CFDC is also recommended [83,100,102].

According to SIMIT and SPILF guideline 2023 mainly “first chooses” antibiotics in different clinical situations were recommended. Several situations were analyzed: VAP due to AmpC β-lactamase-producing Enterobacterales, severe pneumonia due to *P. aeruginosa* resistant to ceftolozane, catheter-related BSI caused by KPC-producing *K. pneumoniae*, severe intra-abdominal infections (IAIs) caused by CRE, ventriculitis and post-neurosurgical meningitis caused by *CR-PA*. The expert decisions of this guideline were based on the necessary balance between antimicrobial stewardship principles, and the need to provide optimal treatment (using pharmacokinetic–pharmacodynamic (PK/PD) modifications and prolonged infusions) for individual patients in each situation [84,104].

Regarding CFDC, it was recommended in combined therapy with an in vitro-active regimen as fosfomycin for severe pneumonia due to *P. aeruginosa* resistant to ceftolozane–tazobactam (with MBL production) [99]. The experts suggested also using β-lactam/β-lactamase inhibitor combination most active in vitro, i.e., imipenem–relabactam, ceftazidime–avibactam, and, in addition (to at least one active in vitro agents), aerosolized colistin or tobramycin [105]. CFDC was also recommended as an alternative regimen in combination with tygecycline or plus fosfomycin and metronidazole for the treatment IAI caused by Enterobacterales MBL (as first choice ceftazidime–avibactam plus aztreonam plus metronidazole was suggested). The results from CREDIBLE-CR study advocate against the use of CFDC as a single agent for the treatment of infections caused by CRAB, so for bloodstream infection (BSI), the panel recommended ampicillin/sulbactam in association with colistin as first choice [84,87,106].

For AMS (antimicrobial stewardship) purposes, the panel suggested considering the CFDC for the treatment of BSI caused by *K. pneumonae* KPC producing (OXA-48, MBL) and DTR-PA. The same guideline, recommended for the treatment of central nervous system (CNS) infection due to CR PA to use ceftazidime–avibactam in combination with fosfomycin as the preferred treatment. The second choice could be either ceftolozane–tazobactam or CFDC in combination with fosfomycin [84,98,107].

## 11. Clinical Trials with Cefiderocol After Its Registration

### 11.1. Efficacy of Cefiderocol Used with Monotherapy

The results of clinical trials confirm that CFDC appears to be a promising therapeutic agent for the treatment of infections caused by CR GNB, even in monotherapy. This was confirmed by A. Karruli et al. [108] in a single-centre, observational, retrospective clinical study (*n* = 28) in which *A. baumannii* was the most frequently found pathogen. Eradication of the causative microorganism was achieved in 14 (77.8%) of the 18 patients in whom follow-up cultures were available. Clinical cure was recorded in 64.3% of patients at 7 days and 50% at 14 days after treatment. There was no effect of CFDC on the development of nephrotoxicity, liver and bone marrow dysfunction. CFDC showed a high safety profile and good efficacy in patients with multiple coexisting MDR infections [108]. Another multicenter, retrospective study by S. Soueges et al. [109] evaluated the efficacy of CFDC treatment in infections caused by MDR GNB. The study included 114 immunocompromised adults treated for MDR infections in 12 French hospitals. CFDC in monotherapy was used in 49.1% of subjects, with a mean duration of therapy of 10 days. At day 28, clinical cure and infection related mortality rates were 53.3% and 25.4%, respectively. By day 28, recurrence was reported in 17.5% of cases. Two subjects developed resistance against *P. aeruginosa* and *S. maltophilia* by day 28 and in three additional cases by day 90. The authors of the study highlighted the promising efficacy of CFDC in the treatment of infections, particularly those with an aetiopathogenesis of S. maltophilia [109].

J. Torre-Cisneros et al. [88], in the PERSEUS study conducted in Spain (*n* = 261), assessed the effectiveness and safety of CFDC in the treatment Gram-negative bacterial infections, excluding *A. baumannii*. The most frequent infections were RTI, IAI and UTI. The most frequent pathogen was *P. aeruginosa*. The study showed a very high clinical cure rate (80.5%), and 28-day all-cause mortality rate 21.5%. In patients with *P. aeruginosa* infection, including with MBLs (42%), the clinical cure rate was even higher 84.5% and 28-day mortality was lower (17.2%). Cefiderocol was well tolerated, adverse drug reactions (ADRs) were observed at 2.2% patients [88].

M. Falcone et al. [110], in an observational retrospective study evaluated the efficacy of CFDC (*n* = 47), compared to colistin (*n* = 77) for CRAB infections (mainly BSI and VAP). There was a higher 30-day mortality rate in patients receiving colistin compared to those receiving CFDC-containing regimens (55.8% versus 34%, *p* < 0.05). Adverse effects, including nephrotoxicity, were more frequent in the colistin group than in the CFDC group. Lack of effective microbial eradication was reported in 17.4% of patients receiving CFDC compared to 6.8% of patients receiving colistin; however, this finding did not reach statistical significance (*p* > 0.05) [110].

Another retrospective study by A. Oliva et al. [111] compared the clinical efficacy of CFDC (*n* = 50) with colistin (*n* = 54) in the treatment of CRAB BSI including patients with septic shock, primary BSI, VAP, catheter-related BSI and HAP. Therapeutic success was higher in the CFDC group than colistin (66% vs. 44.4%, *p* < 0.05). Additionally, mortality at 30 days was significantly lower in patients with HAP/VAP bacteremia on CFDC than on colistin (*p* < 0.05). The incidence of adverse events (mainly acute kidney injury) was higher in the colistin group than in the CFDC group (38.8% vs. 10%, *p* < 0.05). The authors of the study highlighted the therapeutic potential of CFDC in the treatment of CRAB BSI, especially in patients with HAP/VAP bacteremia and patients in a debilitated state, in whom nephrotoxic drugs should be avoided [111]. In a single-center observational study by A. Russo et al. [112] involving cases of VAP bacteremia caused by CRAB in patients with COVID-19, propensity score analysis also showed that regimens containing CFDC and CFDC with fosfomycin in combination were associated with lower 30-day mortality than colistin [112].

### 11.2. The Efficacy of Cefiderocol Used in Combination Therapy

Increasing antibiotic resistance has prompted clinicians to use combination therapy for the treatment of MDR GNB infections, due to the results of studies, mainly in vitro, showing synergistic effects of different antibiotics [113,114]. However, there is no unequivocal confirmation that combination antimicrobial therapy is superior to monotherapy for MDR pathogen infections [114].

M. Piccica et al. [115] evaluated the efficacy of CFDC compared to combination therapy (CFDC and sulbactam or fosfomycin) in infections caused by carbapenem-resistant GNB (mainly *A. baumannii*, *K. pneumoniae*, *P. aeruginosa*) in a retrospective study running from 2021 to 2022 (*n* = 142). The 30-day all-cause mortality rate was 37% (52/142). There was no significant difference in mortality between patients receiving CFDC monotherapy CM (*n* = 70) and those receiving CFDC combination regimen CCR (*n* = 72) (33% versus 40%, respectively). The authors of the study demonstrated that CFDC is an effective treatment option, both in combination therapy and monotherapy for the treatment of CR GN infections [115].

A. E. Ghali et al. [116] demonstrated the potential of CFDC both in monotherapy and in combination (colistin/polymixin B or ceftazidime–avibactam or aminoglycosides) in a multicenter, retrospective study in the control of MDR GNB (*n* = 112). The cohort included patients, with APACHE II score of 15 (19–18). The most common pathogens were *P. aeruginosa* (61/112, 54.5%, of which 55/61 were CR) and CRAB (32/112, 28.6%). Clinical cure was achieved in 68.8% of patients, with a mortality rate of 16.1% and comparable success rates in patients infected with CR GN infections. The incidence of adverse reactions was low; a non-anaphylactic drug-related rash was observed in two patients. Non-susceptibility to CFDC was noted during treatment in six patients, specifically due to *P. aeruginosa* and *A. baumannii* [116].

A multicentre, observational retrospective/prospective study by F. Calò et al. [117] (*n* = 38) found no differences in mortality (51.7 vs. 45.5%) and clinical (41.4 vs. 63.7%) or microbiological (24.1 vs. 9.1%) failure between the CFDC in monotherapy and combination therapy groups in carbapenem-resistant *A. baumannii* (CRAB) infections. The microbiological failure rate after 7 days and at the end of therapy was 20% and 10%, respectively, while the clinical failure rate was 47.5% and 32.5%. The 30-day mortality rate reached 47.5%. The study confirmed the efficacy of CFDC monotherapy as well as in combination therapy [117].

### 11.3. Cefiderocol in the Paediatric Population

There is an increasing number of infections with MDR GNB, including those resistant to carbapenems, among the paediatric population [118,119,120]. Currently, the registered pharmacological agent for the treatment of CRE is ceftazidime-avibactam, while for MDR *P. aeruginosa* it is ceftolozane-tazobactam. The use of CFDCs in children has not yet been regulatory approved [120,121].

J. S. Bradley et al. [120] evaluated the pharmacokinetics, safety and tolerability of CFDC in hospitalized paediatric patients with aerobic GNB infections. The study included 53 patients aged between 3 months and 18 years. Participants received a single dose and multiple doses of CFDC [as a 3 h infusion (every 8 h) at a dose of 2000 mg for body weight ≥ 34 kg and 60 mg/kg for body weight < 34 kg], in terms of renal function. Plasma CFDC concentration profiles were analogous in the single-dose (*n* = 24) and multiple-dose (*n* = 29) groups. The range of minimum concentrations after 8 h was 7.86 to 10.8 μg/mL for single-dose CFDC and 9.64 to 18.1 μg/mL after multiple doses. There were no deaths, development of CFDC-related serious adverse events. The study indicated that steady-state plasma concentrations sustained above critical points of susceptibility of GNB were achieved by multi-dose CFDC administered for 5–14 days, depending on body weight [120].

## 12. Case Reports Assessing the Effectiveness of Cefiderocol

A case report published by Tarski et al. [122] presents a 72-year-old female patient who underwent urgent graftectomy (of a recently transplanted kidney) and was diagnosed with sepsis and intra-abdominal infection caused by *K. pneumonia*-producing NDM and OXA-48. Due to the patient’s severe condition, empirical broad-spectrum antibiotic therapy (meropenem, vancomycin, colistin) was initiated, which was then modified twice, however without any improvement. A culture of purulent material collected during relaparotomy showed that the strain was finally sensitive only to CFDC. Due to no therapeutic effect and the previous lack of access, CFDC was included in monotherapy on the 37th day of hospitalization. After 4 days of treatment, a reduction in inflammation markers and an improvement in the patient’s clinical condition were observed. CFDC therapy lasted for 8 days [122].

La Bella et al. [123]. describe the case of a patient with non-Hodgkin lymphoma who developed aortic endocarditis caused by MDR *A. xylosoxidans*. Two months earlier, the patient had sepsis caused by this bacteria (central line-associated BSI). Despite escalated antibiotic therapy (including carbapenems and colistin), the patient’s symptoms did not subside. Therefore, on the 17th day of hospitalization, CFDC was added to the therapy, leaving fosfomycin, trimethoprim/sulfamethoxazole, and daptomycin. The combination therapy resulted in negative cultures and the resolution of symptoms [123].

The literature also provides an interesting example of the effective use of CFDC in monotherapy in a patient with complex pleural abscess caused by extensively drug-resistant (XDR) *P. aeruginosa*. The patient was admitted to the hospital emergency department with symptoms of respiratory failure in the course of COVID-19 pneumonia and was initially treated with sarilumab, dexamethasone and empirical antibiotics (amoxicillin/clavulanic acid and doxycycline). On the 37th day of hospitalization, the patient developed left-sided empyema and pleural abscess caused by *P. aeruginosa* resistant to ceftazidime/avibactam (MIC 16 mg/L). Due to the persistent abscess CFDC was added together with inhaled colistin, which was discontinued on the 81st day due to a lack of significant therapeutic synergy. After 42 days of CFDC therapy, the patient’s condition stabilized and resulted in patients’ discharge on the 127th day of hospitalization [124].

Among the available sources, we can also find case reports proving the ineffectiveness of CFDC, as in the case of meningitis caused by carbapenem-resistant *A. baumannii*/*calcoaceticus complex* in a 42-year-old female patient. She was initially treated with high doses of ampicillin–sulbactam + CFDC, but after 13 days of therapy, positive cerebrospinal fluid cultures persisted. The treatment was then modified to include sulbactam–durlobactam + meropenem, resulting in a complete cure after 14 days of therapy. During CFDC therapy, a so called paradoxical effect was observed, not previously observed in the disc diffusion test, as well as possible markers of resistance mutations in iron transport genes (*piuA*, *piuC*) and the A515V mutation in PBP3 (the main target of CFDC) [125].

## 13. Alternative Forms of Therapy for Cefiderocol Spectrum Activity

In the conducted systematic literature review, after considering 21 articles published between January 2012 and March 2023, new-generation β-lactamase inhibitors (sulbactam-durlobactam), CFDC, ampicillin–sulbactam and combination therapy, including polymyxin B/colistin, tigecycline, aminoglycosides, carbapenems, fosfomycin and sulbactam were mainly recommended for the treatment of infections caused by MBLs-producing bacteria [126,127,128,129]. However, the European Society of Clinical Microbiology and Infectious Diseases (ESCMID) and the Infectious Diseases Society of America (IDSA), in guidelines for the treatment of infections caused by GN MBL-producing pathogens published in 2022–2023, state that recommendations for carbapenem-resistant *A. baumannii* and *S. maltophilia* are not specific to MBLs-producing pathogens [126].

To date, the combination of ceftazidime-avibactam with aztreonam (CAZ-AVI + ATM) has been the commonly preferred treatment for infections with MBLs strains. Although aztreonam itself is not hydrolyzed by MBLs, it remains active only in about 30% of isolates due to inhibition of its activity by the AmpC and ESBL enzymes frequently coproduced by Enterobacterales MBL(+) strains [130,131]. Therefore, the combination of aztreonam with β-lactamase inhibitors allows for the maintenance of adequate activity due to the efficacy of avibactam against ESBL strains [95,132,133,134]. However, it is worth noting that, despite the absence of reported adverse events, the safety of the above form of therapy is still uncertain. Furthermore, the activity of ATM + CAZ-AVI appears to be limited to selected MBL-producing species due to genotype-determined resistance and the development of additional resistance mechanisms [127]. It is worthy to underline that double carbapenem therapy may offer similar clinical outcomes as ATM + CAZ-AVI for patients with infection caused by *K. pneumoniae* resistant to all available β-lactam/β-lactamase inhibitors [135]. According to the study conducted by A.V. Cienfuegos-Gallet et. al. [136] also the combination MER/VABORBACTAM + ATM was effective for the treatment of infection caused by GNB MBLs strains [136].

It should be underlined that polymyxin antibiotics (colistin and polymyxin B) retain high in vitro activity against many carbapenem-resistant GNB [137]. The prevalence of polymyxin resistance among Enterobacterales producing MBLs was investigated, obtaining 92.6% (*n* = 81) of colistin-sensitive strains and only six isolates resistant to colistin [138]. Some sources report that colistin is now the mainstay of treatment for infections caused by MBLs-producing bacteria [130]. However, due to the increased risk of acute kidney injury (AKI), many cohort studies have suggested not using colistin when there is an alternative therapy option [139,140,141]. It has also been suggested that colistin in monotherapy has less efficacy than its combination therapy, which can also achieve a lower rate of nephrotoxicity [142]. In a study by Falcone et al. [110], colistin-containing regimens were associated with approximately three times higher mortality rates than the CAZ-AVI + ATM, 59.3% and 19.2%, respectively, for CRE producing MBLs. This suggests that polymyxins may be an alternate option to treatment with β-lactams such as CAZ-AVI + ATM [143]. Some in vitro data highlight the potential therapeutic success of using a combination of polymyxin and aztreonam in genotype-specific Enterobacterales [143,144].

Fosfomycin, despite being an antimicrobial agent active against Enterobacterales producing MBLs, has a high risk of acquired resistance [145]. Due to the occurring heteroresistance, fosfomycin monotherapy is not recommended. In the treatment of NDM (+) *K. pneumoniae* infections, fosfomycin should be used in combination therapy preferably with carbapenems and/or colistin with which it shows synergism [146].

Based on an analysis of isolates conducted in November 2018 at the U.S. Centers for Disease Control and Prevention, it was concluded that despite the promising activity of tetracyclines (tigecycline, eravacycline, omadacycline) in vitro, it is recommended that tetracyclines should be used in combination rather than as monotherapy in the treatment of MBLs. Tigecycline alone has an increased mortality rate in monotherapy for MBLs infections, and the discrepancy in doses between EUCAST and FDA causes difficulties in standardising treatment regimens [147,148]. Stable plasmid resistance in blaNDM Enterobacterales treated with tetracyclines has also been described [149].

The in vitro activity of carbapenems against VIM-producing Enterobacterales reaches around 60% [150]. In the absence of sufficient clinical trials and contradictory preclinical data, the risks associated with pharmacotherapy of MBLs infections appear to outweigh the potential benefits, especially when alternative treatment options are present [151].

Aminoglycosides such as amikacin and plasmomycin (accepted only by FDA) are also used in the treatment of infections caused by MBL(+) pathogens. However, in monotherapy they are only recommended for the treatment of UTIs with CRE etiology [152]. For systemic infections, aminoglycoside monotherapy is contraindicated. It is also associated with nephrotoxicity and increasing mortality [153]. Moreover, plasmomycin has significantly reduced activity against *Pseudomonas* spp. and *Acinetobacter* spp. [154]. Given the evidence suggesting increased resistance and adverse events associated with aminoglycoside use, plasmomycin treatment appears controversial [153].

Due to the increasing demand for effective therapeutic options against MBL(+) pathogenes, more advanced pharmaceuticals are being developed. These are combinations of β-lactams with β-lactamase inhibitors targeting MBLs, such as xeruborbactam, as well as those without direct efficacy against MBLs, namely zidebactam and nacubactam [155]. Combinations with in vitro potential against CR Enterobacterales including MBL are: cefepime-taniborbactam, meropenem-xeruborbactam, cefepime-zidebactam and cefepim-nacubactam [137,156]. However, all of the above-mentioned pharmaceuticals are still in the clinical trial phase and are not registered in the US or Europe [157].

Furthermore, it should be emphasized that the therapy of MBLs infections is proving to be a considerable challenge due to the lack of precise dosage, treatment duration and insufficient clinical data [135].

Among the antibiotics used to treat infections with bacteria on the CFDC spectrum, a new drug, a combination of aztreonam and avibactam was approved in the EU in 2024 for the treatment of IAI, HAP and UTI. Resistant only to hydrolysis by MBLs, aztreonam in combination with avibactam that retains activity against strains producing ESBLs and AmpC β-lactamases represents a promising form of therapy for MDR infections of Gram-negative bacteria [95,131,134,158,159].

In addition to CFDC, another attractive form of treatment for carbapenem-resistant *A. baumannii* CRAB infections is FDA-approved (in 2023) sulbactam–durlobactam [160]. It is a combination of a penicillin derivative with a β-lactamase inhibitor active against Ambler class A, C and D serine β-lactamases [161]. It is recommended for use in HAP and VAP [162].

## 14. In Vitro Cefiderocol Activity in Studies Conducted After Its Registration

CFDC has shown strong in vitro activity against carbapenem-resistant Gram-negative pathogens in numerous studies [163,164,165,166]. Galani et al. [163] evaluated the in vitro activities of omadacycline, eravacycline, CFDC, apramycin, and comparator antibiotics against *A. baumannii*. The study lasted six months and included 271 patients from 19 hospitals. CFDC had significant inhibitory activity against 86% of isolates. In addition, the study concluded that CFDC has greater efficacy than minocycline, colistin and ampicillin-sulbactam and eravacycline [163].

Another study by Y. L. Lee et al. [164] analyzed the comparative in vitro activity of CFDC, cefepime–zidebactam, cefepime–enmetazobactam, omadacycline, eravacycline and other agents against carbapenem-non susceptible Enterobacterales (CNSE) (*n* = 201). Analysis showed that CFDC had the highest inhibitory activity against CNSE. Lack of sensitivity to CFDC was observed in only 3.8% (*n* = 1) of *E. coli* isolates and 4.6% (*n*= 8) of *K. pneumoniae* isolates. The study highlighted the promising potential of CFDC against carbapenem-insensitive *E. coli* and *K. pneumoniae* strains [164].

The in vitro activity of CFDC against aerobic GNB pathogens was also assessed in a German study by M. Kresken et al. [165]. It aimed to evaluate the activity of CFDC against two different sets of GNB including ESBL producers and GNB producing different types of carbapenemases (CP). The study revealed high efficacy of CFDC (97.2% and 88.1%) in inhibiting *A. baumannii*, Enterobacterales and *P. aeruginosa*, including CP-producing isolates [165].

In a study by D. Shortridge et al. [166]., the susceptibility of CFDC to clinical isolates of GN that were collected from hospitalized patients in the United States and Europe in 2020 was assessed. Cefiderecol was shown to have the highest antibacterial activity among the agents tested in this study. The susceptibility of Enterobacterales and CRE to CFDC was 99.8% and 98.2%, respectively. The susceptibility of Acinetobacter to CFDC was 97.7%. The susceptibility of *S. maltophilia* to CFDC was 100.0% (CLSI, 2021) and 97.9% (CLSI, 2022) [166].

## 15. Resistance to Cefiderocol in Studies Conducted After Its Registration

Data showed that resistance for CFDC is uncommon according to large multinational cohorts, including isolates resistant to carbapenems, ceftazidime–avibactam, ceftolozane–tazobactam, and colistin. An alarming increase in resistance/non-susceptibility (up to 50%) has been reported in some recent cohorts/data. Several resistance mechanisms leading to resistance to CFDC are enumerated: lactamases, porin mutations, and mutations affecting siderophore receptors, efflux pumps, and target (PBP-3) modifications [31,167].

In an analysis of 78 studies covering 82,035 clinical isolates conducted by S. Karakonstansis et al. [168], the incidence of resistance to CFDC was assessed depending on the bacterial species. The level of resistance was mentioned as low, at *S. maltophilia* 0.4%, *P. aeruginosa* 1.4%, *A. baumannii* 8.8%. Among carbapenem-resistant strains, the incidence of resistance was slightly higher, at 12.4% for CR Enterobacterales and 13.2% for CR *A. baumannii*. Among bacteria producing New Delhi MBL, 38.8% of Enterobacterales and 44.7% of *A. baumannii* were insensitive to CFDC [168].

A systematic review including articles published up to May 2022 in PubMed and Scopus summarized the involvement of β-lactamases (NDM, AmpC), PBP-3 protein modifications, porin mutations and siderophore receptors in generating resistance to CFDC and showed that *A. baumannii* was particularly heteroresistant [167]. Although the complex resistance to CFDC was not sufficiently characterized, mutations of the TonB-dependent iron transporter pathway genes played a role [169,170]. The mutations resulted in reduced activity of the pathway, leading to an increase in the Minimal Inhibitory Concentration (MIC) of the siderophore antibiotic CFDC. In the case of *A. baumannii*, overexpression of the iron transporter regulator operon FecIRA was associated with at least a fourfold increase in MIC [171,172]. Meanwhile, in *P. aeruginosa* as much as a 32-fold increase in MIC associated with deletion of the *piuD* gene was observed [173].

Furthermore, Ito et. al. [70] analyzing the in vitro antimicrobial properties of CFDCs, found that *cirA* and *fiu* deletions led to a 16-fold increase in the MIC of CFDCs. Mutation of the *BaeS* gene also significantly affected the resistance of *K. pneumoniae* to CFDC [174,175].

The ampC gene encodes the AmpC β-lactamase. Mutations of the enzyme observed in *E. cloacae* and *E. hormaechei* resulted in conformational changes in the protein limiting the sensitivity of the strains to this antibiotic [176].

A study by K. M. Kazmierczak et al. [76] involving 151 CRE strain isolates showed that CFDC was active against 100% of KPC-producing Enterobacterales strains, but only in 58% of NDM (+) cases. Despite the clearly observable phenomenon of CFDC MIC creep due to the following mutations, it is not known whether the changes remaining in sensitivity have significant clinical implications [76].

Although in vivo resistance to CFDC has rarely been observed in clinical trials, the available case reports confirm its occurrence [23].

All studies discussed throughout this review were included in the synthesis, and their main results are presented in Table 3.

## 16. Study Limitations

Most studies suggest significant efficacy of CFDC in treating MDR GNB infections; however, small sample sizes and population heterogeneity limit the ability to draw firm conclusions. This heterogeneity was due to differences in the study populations (age, gender, health status), study errors (different measurement methods, study duration), and different outcome classification principles. Further studies using standardized criteria and larger cohorts are necessary to fully determine the therapeutic potential of CFDC

## 17. Conclusions

CFDC is an innovative siderophore cephalosporin approved by the EMA for the treatment of infections caused by aerobic Gram-negative bacteria in adults with limited treatment options. Analysis demonstrated its significant efficacy across a wide range of in vitro and in vivo studies against MDR GNB, including CRPA, CRAB, and CRE (WHO Critical Priority Pathogens). Clinical studies have shown CFDC to be an effective drug with few adverse effects. Furthermore, nonclinical safety data regarding mutagenicity and teratogenicity did not reveal a risk to humans. When used CFDC appropriately within antibiotic stewardship guidelines, this drug is an effective, well-tolerated targeted treatment option for patients with severe clinical conditions.

## Figures and Tables

**Table 1:** Recommended dosage of CFDC, depending on creatinine clearance, in patients with renal function shown as creatinine clearance (CLcr) < 60 mL/min and patients receiving intermittent HD [51,52].

Renal Function Shown as Creatinine Clearance	Dosage	Frequency
30–59 mL/min	1.5 g	every 8 h
15–29 mL/min	1 g	every 8 h
<15 mL/min and patients undergoing intermittent HD	0.75 g	every 12 h

**Table 2:** Recommended dosage of cefiderocol in patients undergoing continuous renal replacement therapy (CRRT) depending on the effluent flow rate [53].

Effluent Flow Rate in Continuous Renal Replacement Therapy	Dosage
≤2 L/h	1.5 g every 12 h
2.1–3 L/h	2 g every 12 h
3.1–4 L/h	1.5 g every 8 h
≥4.1 L/h	2 g every 8 h

**Table 3:** Summary of research results on cefiderocol efficacy.

	Study Type/Number of Included Patients	Type of Infection	CFDC Monotherapy	CFDC Combination Therapy	Comparator	Pathogen	CFDC Cure Rate/Microbiological Eradication (%)	CFDC Clinical Improvement (%)	Comparator Cure Rate/Microbiological Eradication (%)	Comparator Clinical Improvement (%)	Other Aim/Results	Author of the Study
1	Prospective, Phase I Pharmacokinetic Study In Healthy Volunteers (*n* = 20)	(Not Applicable)	Yes	No	None	(Not Applicable)	(Not Applicable)	(Not Applicable)	(Not Applicable)	(Not Applicable)	This study assessed intrapulmonary penetration after a single intravenous dose of CFDC (2000 mg infused over 60 min) in healthy adult males. The study found good CFDC pulmonary penetration. CFDC penetrates into ELF, and ELF and plasma concentrations appear to be parallel.	T. Katsube et al. [66]
2	Prospective, Phase I Pharmacokinetic Study (single-arm, open-label) (*n* = 38)	(Not Applicable)	Yes	No	None	(Not Applicable)	(Not Applicable)	(Not Applicable)	(Not Applicable)	(Not Applicable)	The pharmacokinetics and safety of CFDC in subjects with renal impairment were assessed following a single 1000 mg intravenous 1-h infusion of CFDC. The protein-unbound fraction was similar between various renal function groups. Renal impairment impacted AUC, CL, and t 1/2 without affecting Cmax.	T. Katsube et al. [65]
3	In Vitro (preclinical, laboratory)	Different types of infection caused by GNB	No	No	Ceftazidime, Cefepime, Meropenem, Piperacillin/ Tazobactam, Colistin	*Pseudomonas aeruginosa Acinetobacter baumannii Enterobacteriaceae* (including *Klebsiella pneumoniae*, *Escherichia coli*), *Stenotrophomonas maltophilia*	(Not Applicable)	(Not Applicable)	(Not Applicable)	(Not Applicable)	The study assessed MICs for CFDC and other B-lactam antibiotics against GNB, aerobic bacteria and GNB with betalactamases. The study showed that CFDC has potent in vitro activity against a broad range of aerobic GNB, including Enterobactariacae and non-fermenting MDR strains. This study also revealed that iron transporters such as PiuA of *P. aeruginosa* and CirA and Fiu of *E. coli* are involved in the permeation of cefiderocol into bacterial cells.	Ito et al. [70]
4	Part Of The Sidero-wt-2014 In Vitro Surveillance Study	Hospital -acquired infection (Clinical isolates of MDR and CR-GNB)	No	No	Meropenem Cefepime Ceftalozone/tazobactam Ceftazidime/awibaktam Ciprofloxacin Colistin	*Enterobacteriaceae*, *Pseudomonas aeruginosa*, *Acinetobacter baumannii*	(Not Applicable)	(Not Applicable)	(Not Applicable)	(Not Applicable)	The in vitro study assessed MIC’s for CFDC and other antibiotics against GNB. CFDC showed high in vitro efficacy against CR strains, including strains with serine carbapenemases and MBL’s and carbapenemase-negative meropenem-non- susceptible GNB isolates (also betalactamases ESBL+ and AmpC) of *Enterobacteriaceae*, *P. aeruginosa* and *A. baumannii* collected as part of a global surveillance program. CFDC was also active against colistin-resistant isolates of *Enterobacteriaceae*, including isolates carrying the transmissible colistin resistance determinant, mcr-1.	K. M. Kaźmierczak et al. [76]
5	Observational, Retrospective- prospective (pilot study) (*n* = 27)	PN, SSTI, IAI, UTI , bacteremia	Yes	No	None	Carbapenem-resistant *Acinetobacter baumannii* (CRAB) 59% of strain were CFDC heteroresistant −30% from Italy, 73% from USA	(Not Applicable)	(Not Applicable)	(Not Applicable)	(Not Applicable)	28-day clinical success 30%, survival rate 73%; failure rates was 81% (CFDCMIC ≤ 0.5 mg/L vs. 55% (CFDC MIC ≥ 1); clinical failure was 81% (CFDC heteroresistant) vs. 55% (CFDC susceptible)	R. K. Shields et al. [77]
6	Prospective, Randomized, Double-blind, Phase III Non-inferiority Study (*n* = 300)	HAP/VAP/HCAP	No	Yes (100%) Linezolid- to ensure coverage of Gram-positive bacteria	Meropenem	Gram-negative pathogens likely susceptable to meropenem	Clinical cure rate 65; Microbiological eradication 41	(Not Applicable)	Clinical cure rate 67; microbiological eradication 42	(Not Applicable)	14-day all-cause mortality was (ACM) (CFDC 12.4% vs. MER 11.6%, *p* = 0.002)	R. G. Wunderink et al. [78]
7	Prospective, Randomized Open-label, Multicenter, Descriptive, Phase III (*n* = 118)	HAP/VAP(NP)/ HCAP/BSI/sepsis /cUTI	Yes (85%)	Yes (15%) CFDC + 1 from: Tigecycline TMP-SMX Fosfomycin Fluoroquinolones (ciprofloxacinlevofloxacin)	Best Available Therapy (BAT)	Carbapenem-resistant Gram-negative pathogens	Cure rate (7 days [+/−2] after the end of treatment) NP 50 BSI 43 Microbiological eradication UTI 53		Cure rate (7 days [+/−2] after the end of treatment) NP 53 BSI 43 Microbiological eradication UTI 20		28-day ACM was (CFD 25% vs. BAT 18%); and of study ACM (CFDC 34% vs. BAT 18% for NP)	M. Bassetti et al. [79]
8	Multicentre, Double-Blind, Phase 2 Study (*n* = 452)	cUTI with/without acute pyelonephritis	Yes (67%)	None	Imipenem–Cilastatin (23%)	Gram-negative pathogens likely susceptible to imipenem	Clinical cure 90	73	Clinical cure 87	56	Clinical and microbiological response was (CFDC 73% vs. IMP 55%, *p* = 0.004)	S. Portsmouth [80]
9	Single-Centre, Retrospective Observational Study (*n* = 28)	PN/BSI/UTI/IAI	Yes	None	None	45 Gram-negative Isolates, most common: *Acinetobacter baumannii* (31.1%)	Clinical cure 68 (on a basis treatment failure); Microbiological eradication 77.8	64.3 at day 7; 50 at day 14	(Not Applicable)	(Not Applicable)	Treatment failed 32%	A. Karruli et al. [108]
10	Retrospective Multicentre Observational Study of Immunocompromised Patients (*n* = 114)	RTI, UTI, CR-BSI, SSTI, CNSI	Yes (49.1%)	Yes (50.9%)	None	Multidrug-resistant Gram-negative Bacteria (various Species), most common was *P. aeruginosa* 56%	Clinical cure 53.3%	(Not Applicable)	(Not Applicable)	(Not Applicable)	28-day overall mortality was 37.7%, attributable mortality 25.4%; 90-day overall mortality 52.2%, attributable mortality 35.1%	S. Soueges et al. [109]
11	Retrospective Single-Centre Observational Cohort Study (*n* = 124)	BSI, VAP, SSI	Yes (31.9%)	Yes (68.1%) CFDC + 1 from others Tigecycline: Fosfomycin: Ampicillin/sulbactam Meropenem/Vaborbactam	Colistin-containing Regimens (15.6% in monotherapy)	Carbapenem-resistant *Acinetobacter baumannii* (CRAB), at 23% of patients infection was polymicrobial	(Not Applicable)	(Not Applicable)	(Not Applicable)	(Not applicable)	30-day mortality was COL 55% vs. CFDC 34%, *p* = 0.018; microbiological failure 17.4%CFDC vs. 6.8% COL, *p* = 0.079	M. Falcone et al. [110]
12	Retrospective Single-Center Observational Cohort Study (*n* = 104)	BSI (source: CRBSI, primary BSI, VAP, HAP)	Yes (13%)	Yes (35%) Cfdc + Ampicillin/sulbactam Cfdc + Fosfomycin	Colistin-containing Regimens (52%)	Carbapenem-resistant *Acinetobacter baumannii* (CRAB), at 21% of patients infection was polymicrobial	Clinical cure 66 clinical cure at VAP 70 microbiological eradication 72	64	Clinical cure 44.4 clinical cure at VAP 10 microbiological eradication 66	46.3	Overall mortality CFD vs. COL was 57.1% vs. 61.1%, *p* = 0.682); 7 day mortality 16% vs 20.4%, 14 day mortality (22% vs. 31.5%, *p* = 0.276), adverse events (10% vs. 38.8%, *p* < 0.001)	A. Oliva et al. [111]
13	Observational Single-Center Real-Life Study at Patients with COVID 19 (*n* = 73)	VAP + BSI	No	Yes (100%) CFDC + 1 or more from others Fosfomycin, Tigecycline, Meropenem, Trimetoprime/sulfamethoxazole	Colistin-containing Regimens (22.2%in monotherapy)	Carbapenem-resistant *Acinetobacter baumannii* (CRAB)	(Not Applicable)	(Not Applicable)	(Not Applicable)	(Not Applicable)	ACM at day 14 CFD 5.2% vs. COL 75.9%, *p* < 0.001; ACM at day 30 CFD 31.5% vs. COL 98.1%, *p* < 0.001 CFDC and CFDC +Fosfomycine combined therapy were independently associated with 30 day survival.	A. Russo et al. [112]
14	Retrospective Tree-Centre Cohort Study (*n* = 142)	bacteraemia, UTI, LRTI, IAI, ABSSSI	Yes (49%)	Yes (51%)	None	*Acinetobacter baumannii*, *Klebsiella pneumoniae*, *Pseudomonas aeruginosa* and other Pathogens	difference in microbiological cure between CFDC in monotherapy and combination therapy: no significant difference was found 45.8% vs. 52.3%, *p* = 0.68	(Not applicable)	(Not Applicable)	(Not Applicable)	30-day ACM was 37%; difference in 30-day mortality between CFDC in monotherapy and combination therapy was no significant 32.9%% vs. 40.3%, *p* = 0.39; no association between bacterial species and mortality was found	M. Piccica et al. [115]
15	Multi-center, Retrospective Cohort Study (*n*= 112)	VAP, HAP, BSI, SSI, UTI, intra-abdominal infection and osteomyelitis	Yes	No	None	*Pseudomonas aeruginosa*, Carbapenem-resistant *Acinetobacter baumannii*, *Klebsiella pneumoniae*, *Escherichia coli*, *Stenotrophomonas maltophilia*, *Achromobacter xylosoxidans*	(Not Applicable)	68.8	(Not Applicable)	(Not Applicable)	30-day mortality was 16.1%; clinical worsening 14.3%, 30-day microbiological recurrence was 14.3%; adverse events: 2/112 patients experienced a non anaphylactic rash; 6/112 patients developed on treatment CFDC resistance	A. E. Ghali et al. [116]
16	Multicenter Observational Retrospective/prospective Study (*n* = 38)	BSI, pneumonia, UTI, skin and soft tissue infection, intra-abdominal infection, bone infection	Yes (72.5%)	Yes (27.5%) CFDC + one from others ampicillin/sulbactam, fosfomycin, tigecycline, amikacin	None	Carbapenem-resistant *A. baumannii* (CRAB)	67.5% on a basis clinical failure 32.5%	90% on a basis microbiological failure 10%	(Not Applicable)	(Not Applicable)	30-day mortality was 47.5%; clinical failure 32.5%, microbiological failure 10%; no significant difference in mortality was observed between combined therapy and monotherapy (51.7% vs. 45.5%) as well as in clinical failure 41.4% vs. 63.7%	F. Calo et al. [117]
17	International, Multicenter, Open-label Phase 2 Study at pediatric patients (*n* = 53)	complicated UTI, complicated intra-abdominal infection, HAP/VAP and sepsis or BSI	Yes (single dose: 45%; multiple dose: 55%)	No	None	Gram-Negative Bacteria	(Not Applicable)	(Not Applicable)	(Not Applicable)	(Not Applicable)	The study investigated the pharmacokinetics, safety and tolerability of single-dose and multiple-dose CFDC. Plasma concentration profiles were similar with single-dose and multiple-dose of CFDCc. No serious adverse events were observed.	J. S. Bradley et al. [120]
18	Case Report	Complicated Hospital-Acquired Intraabdominal Infection	Yes	No	No (earlier therapy: Meropenem + Vancomycin + colistin; Aztreonam + meropenem/vaborbactam + colistin And Tigecycline; Ceftazidime/awibaktam + Aztreonam)	*K. pneumoniae* Ndm, Oxa 48	Yes/Yes	After 4 days of CFDC monotherapy the Inflammatory markers significant decreased and improvement in clinical state was observed	(Not applicable)	(Not applicable)	The study showed clinical success after CFDC application against *K. pneumonie* NDM, OXA 48 responsible for cIAI.	Tarski et al. [122]
19	Case Report And Literature Review	Aortic Endocarditis	No	Yes (fosfomycin, trimethoprim/sulfamethoxazole and daptomycin)	No (earlier therapy) piperacillin/tazobactam and teicoplanin followed by imipenem; meropenem, fosfomycin, trimethoprim/sulfamethoxazole and daptomycin	*Achromobacter xylosoxidans*	(Not Applicable)	After introducing CFDC, the bactericidal activity of the serum increased from no activity to a dilution of 1:8 (trough sampling) and 1:32 (peak sampling)	(Not Applicable)	(Not Applicable)	The study showed clinical success after CFDC treatment against *Achromobacter xylosoxidans*	La Bella et al. [123]
20	Retrospective Multicentre Study (*n* = 261)	RTI, IAI, UTI	Yes (65.1%)	Yes (34.9% colistin mainly)	none	Gram-negative bacteria except *A. baumannii*	80.5 *P. aeruginosa* clinical cure 84.5	(Not Applicable)	(Not Applicable)	(Not Applicable)	28-day mortality was 21.5%, 28-day mortality for *P. aeruginosa* was 17.2%; adverse events was found et 7/261 patients	J.Torre -Cisneros et al. [88]
21	Retrospective, Multicentre In Vitro Surveillance Study (*n* = 271)	Bacteremia	No	No	Apramycin (EBL-1003), Minocycline, Colistin and Ampicillin-sulbactam, Eravacycline, Tigecycline	*A. baumannii* (98% with carbapenemase OXA 23)	(Not Applicable)	(Not Applicable)	(Not Applicable)	(Not Applicable)	The study compared the in vitro activity of new and conventional antibiotics against *A. baumannii* strains from bacteremia. Cefiderocol showed the highest activity among actually available/registered drugs tested. CFDC exhibited 86% isolates, apramycin 100%, monocycline, colistin, ampicillin/sulbactam < 19% according to EUCAST breakpoints. According to CLSI breakpoints CFDC exhibited 93.4% isolates *A. baumannii*.	Galani et al. [163]
22	Multicentre in In Vitro Surveillance Study	Different types of infections caused by Enterobacterales carbapenem non susceptible (CNSE)	No	No	Cefepime/zidebactam, Cefepime/enmetazobactam, Ceftazidime/awibaktam, Omadacycline, Eravacycline	*Enterobacteriales* Carbapenem non susceptible	(Not Applicable)	(Not Applicable)	(Not Applicable)	(Not Applicable)	The study evaluated the in vitro activity of new antibiotics against CNSE. Cefiderocol showed the highest activity (>95%). Only 3.8% Of *E. coli* Isolates And 4.6% of *K. pneumoniae* Isolates Were Not Susceptible To Cfdc/ *E. coli*: 96.2% susceptible *K. pneumoniae*: 95.4% susceptible	Y.L. Lee et al. [164]
23	Multicentre In Vitro surveillance study	hospital-acquired infections (clinical isolates of GNB including CR strains)	No	No	None	*Enterobacteriales*, *Pseudomonas aeruginosa*, *Acinetobacter baumannii*	(Not Applicable)	(Not Applicable)	(Not Applicable)	(Not Applicable)	The study assessed in vitro activity of CFDC against GNB. Study showed very high susceptibility for CFDC: Enterobacterales 97.2%, carbapenemase producing isolates 88.1%, Assessed MICs for: *Enterobacterales* MIC_50_/_90_ = 0.12/1.0 mg/L *P. aeruginosa*: MIC_50_/_90_ = 0.12/0.5 mg/L *A. baumannii*: MIC_50_/_90_ = 0.06/0.12 mg/L	M. Kresken et al. [165]
24	Multicentre In Vitro Surveillance Study	hospital-acquired infections (clinical isolates of GNB including MDR and CR strains)	No	No	Amikacin, Cefepime, Ceftazidime, Ceftazidime/awibaktam, Ciprofloxacin, Colistin, Imipenem/relebactam, Meropenem, Meropenem/vaborbactam, Piperacillin/tazobactam, Tigecycline	*Enterobacteriales*, *Pseudomonas aeruginosa*, *Acinetobacter* spp., and *S. maltophilia I*	(Not Applicable)	(Not Applicable)	(Not Applicable)	(Not Applicable)	The study evaluated in vitro activity of CFDC and other antibiotics against GNB. CFDC showed the highest activity among all antibiotics tested. Susceptibility for CFDC: Enterobacterales 99.8%; *P. aeruginosa* 99.6%; *Acinetobacter* spp. 97.7%; *S. maltophilia* 100.0% (CLSI, 2021) and 97.9% (CLSI, 2022)	D. Shortridge et al. [166]
25	Systematic Review and Meta-Analysis In Vitro Studies	(Not Applicable)	(Not Applicable)	(Not Applicable)	(Not Applicable)	*Enterobacterales*, *Pseudomonas aeruginosa*, *Acinetobacter baumannii*, *Stenotrophomonas maltophilia*	(Not Applicable)	(Not Applicable)	(Not Applicable)	(Not Applicable)	The study showed that overall resistance to CFDC was low, but in some regions and among carbapenem-resistant strains, it was significantly higher: *S. maltophilia*: 99.6% susceptible (0.4% CFDC-NS, 95% CI 0.2–0.7%) *Enterobacteriales*: 97.0% susceptible (3.0% CFDC-NS, 95% CI 1.5–6.0%) *P. aeruginosa*: 98.6% susceptible (1.4% CFDC-NS, 95% CI 0.5–4.0%) *A. baumannii*: 91.2% susceptible (8.8% CFDC-NS, 95% CI 4.9–15.2%)	S. Karakostansis et al. [168]

## Data Availability

No new data were created or analyzed in this study.
